# A Pilot Study of Gene Expression Analysis in Peripheral Blood Mononuclear Cells in Response to a Hypocaloric Mediterranean Diet

**DOI:** 10.1155/2022/3706753

**Published:** 2022-01-11

**Authors:** Daniel de Luis, David Primo, Olatz Izaola, Rocío Aller

**Affiliations:** Center of Investigation of Endocrinology and Nutrition, Medicine School and Dept of Endocrinology and Nutrition, Hospital Clinico Universitario, University of Valladolid, Valladolid, Spain

## Abstract

**Background:**

Few studies have examined gene expression in peripheral blood mononuclear cells (PBMCs) after a dietary intervention.

**Objective:**

Our study is aimed at evaluating in a pilot study the peripheral blood gene expression in obese patients after weight loss secondary to a hypocaloric Mediterranean diet.

**Design:**

A sample of 11 obese subjects without metabolic syndrome was enrolled. Biochemical, anthropometric parameters and microarray analysis were performed at baseline and after 6 months of dietary intervention.

**Results:**

The mean age was 43.1 ± 6.3 years, and the mean body mass index (BMI) was 38.6 ± 8.1 kg/m^2^. All the next improvements were statistically significant: body weight −7.4 ± 1.9 kg, BMI -2.5 ± 0.2 kg, fat mass −5.7 ± 1.2 kg, waist circumference −5.8 ± 1.2 cm, triglycerides −17.4 ± 6.5 mg/dl, C-reactive protein −3.1 ± 1.5 mg/dL, insulin −2.1 ± 1.0 mUI/L, and HOMA-IR −0.7 ± 0.2 units. We identified 634 differentially expressed genes: 262 genes with relative higher expression levels and 372 with lower expression levels. Cluster analysis showed 35 genes in nutritional disease and 17 genes in endocrine system. The most relevant gene was thyroid peroxidase (TPO), and this gene was overexpressed, and the next genes carbonic anhydrase VI (CA6), caveolin protein 1 (CAV1) and solute carrier family type 12 (SLLC12A3), soluble carrier family type 12 (SLLC12A3), beta 3 receptor (ADRB3), and glutamate receptor ionotropic N methyl D aspartate 2 A (GRIN2A) were all underexpressed.

**Conclusion:**

In PBMC from obese patients after a diet with a Mediterranean pattern, the expression of 634 genes, of the endocrine system and of nutritional disease, is modified.

## 1. Introduction

Obesity is an important health problem in our countries, producing an important morbidity from metabolic syndrome, diabetes mellitus type 2, cardiovascular risk, and malignant tumors [1]. This high prevalence of obesity is secondary to an excessive calorie intake and low physical activity in our society. Moreover, some evidence also indicates an important genetic contribution to adiposity [2]. Taking into account these data, the evaluation of gene profile with mRNA expression is being performed by microarrays [3]. Based on this, gene profile designs in human being have been performed on adipocytes, truly on subcutaneous adipocytes [4] and evaluation of visceral adipocytes is realized in bariatric obese subjects [5]. However, it is impossible to perform gene expression studies in real life by submitting patients to biopsies, and for this reason, it is necessary to evaluate the obtaining of genetic material from other more easily accessible tissues [6]. Peripheral blood mononuclear cells (PBMCs) have been besought as a device to understand different areas, for example, cancer [7] and heart attacks or strokes [8]. In this topic area, a huge agreement (80-90%) of gene profile between PBMC and other cells has been reported [9]. Thus, transcriptome evaluation of PBMC is an interesting area for evaluating responses to different therapies.

Gene expression changes to dietary modifications have frequently been evaluated in subcutaneous adipose tissue [10]. Few studies have examined gene expression in blood after a dietary intervention [11–14]. And even fewer are those who have studied the effect of a diet with a Mediterranean profile [15–17], without having evaluated obese patients without cardiovascular risk in these studies. One of the dietary patterns with the greatest beneficial effect on biochemical parameters after weight loss is the Mediterranean diet pattern [18, 19]. The Mediterranean dietary pattern has demonstrated multiple cardio metabolic improvements such as lipid control and glucose improvement [20].

The objective of our design was to evaluate the PBMC gene expression in obese subjects of low cardiovascular risk after weight change secondary to a hypocaloric Mediterranean diet during 6 months.

## 2. Materials and Methods

### 2.1. Subjects

This pilot design was realized according to the Declaration of Helsinki, and all procedures with human beings were passed by the HCUVA Ethics Central Committee. A group of 11 obese subjects without metabolic syndrome was recruited. These obese subjects were recruited in a Nutrition Board. All subjects gave signed consent. In sedentary obese subjects, inclusion criteria were the following: age range (30-60 years) and body mass index > 30 kg/m^2^, and the next exclusion criteria were the following: previous heart attack or stroke and alteration in glucose metabolism such as fasting plasma glucose > 110 mg/dl or presence of metabolic syndrome (MS) categorized by the criteria of the ATPIII [21].

### 2.2. Procedures

Fasting (8 h) glucose levels, C-reactive protein (CRP) levels, insulin levels, calculated insulin resistance (HOMA-IR), and lipid profile (LDL cholesterol, HDL cholesterol, and plasma triglycerides) were determined at basal and after 6 months of intervention. Height, weight waist circumference, and body mass index were performed in both times, too. An impedance bioelectric was measured in order to determine fat mass. All these determinations were performed at the same time of the day (morning). Microarrays from PBMC were realized at both times, too.

### 2.3. Dietary Change

The diet change was a hypocaloric diet with a Mediterranean style (1508 kcal per day) (6 months). This caloric intake was calculated by subtracting 500 calories from the caloric intake obtained with the Harris Benedict formula in our sedentary obese population (2039.4 ± 93.7 kcal per day). The ratio of nutrients was the following: 25% from lipids and 23% from proteins, and the main nutrient was 52% from carbohydrates. The range of fats was as follows: 50.7% of monounsaturated dietary fats, 38.5% of saturated dietary fats, and 11.8% of polyunsaturated dietary fats. Diet contained with extravirgin olive oil (an amount of 30 ml/day) (OliDuero, Matarromera, Sl), 3 portions of fish per each week, 3 portions of nuts per a week, and vegetables and fresh fruits 4-5 portions each day. The monitoring of the intervention was realized each 2 weeks by a dietitian. All subjects received briefing to impress their intakes for three different days. Records were evaluated by a registered dietitian with a computer program [22].

### 2.4. Biochemical Assays

Biochemical measurements, including glucose, insulin, CRP, and lipid profile, were realized with COBAS INTEGRA 400-Analyser (Roche Diagnostic, Basel, Switzerland). LDL cholesterol was calculated with the well-known Friedewald formula (LDL cholesterol = total cholesterol − HDL cholesterol − triglycerides/5) [23]. Based on these parameters, insulin resistance (HOMA-IR) was calculated with homeostasis-model-assessment for using the next parameters (glucose × insulin/22.5) [24].

### 2.5. Anthropometric Measurements

Corporal weight was determined to an accuracy of 100 g, and BMI calculated as body weight/(height^2^). Waist circumference and hip circumference were measured, too. Waist to hip ratio (WHR) was calculated with the ratio waist/hip circumference. Impedance bioelectric was used to calculate body composition [25]. The equation of this device was used: (0.756 height^2^/resistance) + (0.110 × body mass) + (0.107 × reactance)–5.463.

### 2.6. Microarrays Assays

2.5 ml of blood was obtained with PaxGene laboratory tubes (Becton Dickinson, USA). Total RNA was obtained from blood cells using the PAXgene Blood RNA System (PreAnalytix, Geneva, Switzerland). RNA was measured by RNA 6000 Nano Bioanalyzer (Agilent Technologies, LA, CA, USA) assay. Around 2000 ng of each RNA sample was evaluated with the RNeasy MinElute Cleanup kit (QIAGEN, Hilden, Germany). RNA was obtained with 20 ml of RNase-free water. 150 ng of RNA was utilized to produce Cyanine 3-CTP-labeled cRNA with (Agilent p/n 5190-0442). Following “Single-Color Microarray-Based Gene Profile Analysis” protocol Version 5.8 (Agilent p/n 4140-90040), 4 *μ*g of cRNA was hybridized with Genome Oligo Microarray Kit (Agilent p/n G2519F-014850) with 41,000+ human genes and transcripts. These arrays were evaluated in an Agilent Microarray Scanner (Agilent G2565BA), and parameters were extracted using Agilent Feature Extraction Software 9.5.3.

These microarrays datasets were located at the repository (E-MTAB-3017). Modifications in microarray gene profile were evaluated for 2 genes of our experiment by qPCR, according to the manufacturer (Real-time Ready, Roche Applied Science, Germany). Data evaluation was performed with the GeneSpring GX 11.0 software. The natural data was cleansing and normalized. The modification of the data was performed with the average of all samples. Quality of the results was realized on principal component analysis plots. All the 22 arrays reached these standards and were included in the evaluation. In order to obtain differences expressed between groups at the level of significance *p* < 0.05, the Mann-Whitney test was used with Benjamini-Hochberg corrections and finally a fold change = +2 as threshold was used. To evaluate the function, determinations were performed with the Ingenuity Pathway Analysis 8.5 software (IPA). GeneSpring GX 11.0 was utilized for evaluating gene hierarchical clustering.

### 2.7. Statistical Analysis

A descriptive analysis of the data (mean, standard deviation, and frequency) and subsequently an inferential analysis were carried out. Each variable was examined for normality with the Kolmogorov–Smirnov test. For descriptive purposes, results were expressed as average ± standard deviation. In within-groups, we conducted paired *t*-tests for biochemical parameters at baseline and after caloric restriction. In between-groups, independent *t*-test was used to compare the differences in both times. Nonparametric variables were analyzed with the Mann-Whitney *U* test. Categorical variables were analyzed with the chi-square test, with Yates correction as necessary, and Fisher's test. IBM SPSS version 23.0 software package (SPSS Inc. Chicago, IL) was used to make the statistical analyses. Significance was assumed for *p* < 0.05.

## 3. Results

Eleven obese subjects (8 females and 3 males) signed informed consent and were recruited in the design. The average age was 43.1 ± 6.3 years, and the basal average BMI was 38.6 ± 8.1 kg/m^2^.

After 6 months of diet, all obese subjects achieved dietary goals (1503.1 ± 287.1 kcal/day). The average intake of macronutrients at 6 months was the following: 50.4% from carbohydrates, 25.2% from fatty acids 50.1% from monounsaturated fatty, 37.9% from saturated fatty acids, 13.0% from polyunsaturated fatty acids, and 24.4% from proteins.

Anthropometric and biochemical parameters of subjects at initial time and after 6 months of dietary change are reported in Table 1. BMI, body weight, fat mass by impedance, waist circumference, ratio waist/hip (WHR), triglyceride, CRP, insulin, and HOMA-IR decreased. All next improvements were statistically significant: body weight −7.4 ± 1.9 kg, BMI −2.5 ± 0.2 kg, fat mass −5.7 ± 1.2 kg, WHR −0.02 ± 0.001 cm, waist circumference −5.8 ± 1.2 cm, triglycerides −17.4 ± 6.5 mg/dl, CRP −2.1 ± 1.5 mg/dL, insulin −2.1 ± 1.0 mUI/L, and HOMA-IR −0.7 ± 0.2 units.

**Figure 1:**
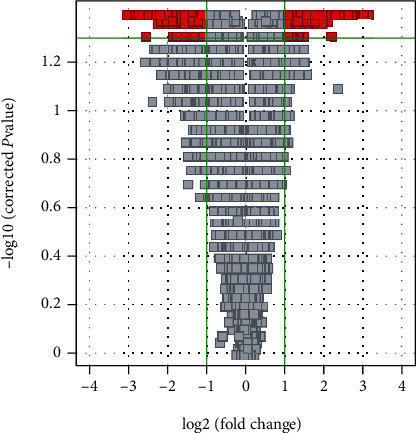
Volcano plot analysis applied to the microarray data revealed 634 genes that were significantly expressed (*p* < 0.01 with fold change 1.5 (up or down)) in basal obese patients compared to postintervention obese patients. The plot shows a log2-fold change in mRNA expression between the 2 groups (pre- and postdietary intervention) on the *X*-axis and the negative log of *t*-test *p* values on the *Y*-axis. Each gene was represented by a single point, and dark gray areas indicate genes with significant changes in gene expression.

**Figure 2:**
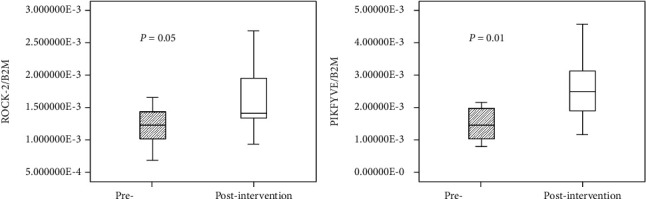
Validation of the results from microarrays by RT-PCR assay. Mann-Whitney test was used to compare gene expression levels between pre- and postintervention at the level of significance *p* < 0.05.

The volcano figure evaluation demonstrated changes in mRNA profile between initial time and after 6 months of dietary intervention considering FDR revised *p* value cutoff < 0.05 (downregulated) and FD cutoff = 2 (upregulated). Our data detected 634 expressed genes with a significant difference: a total of 262 genes showed a higher expression levels and a total of 372 a lower expression levels (Figure 1). The most important of these different expressed genes in PBMCs showed expression levels lower than 2.5-FC (up or down) when were compared with preintervention values, and some genes reported FC ≥ 2.5. Modifications in gene profile detected by microarrays were verified for 2. Gene expression of PtdIns3 P 5-kinase and rho-kinase II determined by quantitative RT-PCR was used with Mann-Whitney test (Figure 2).

**Figure 3:**
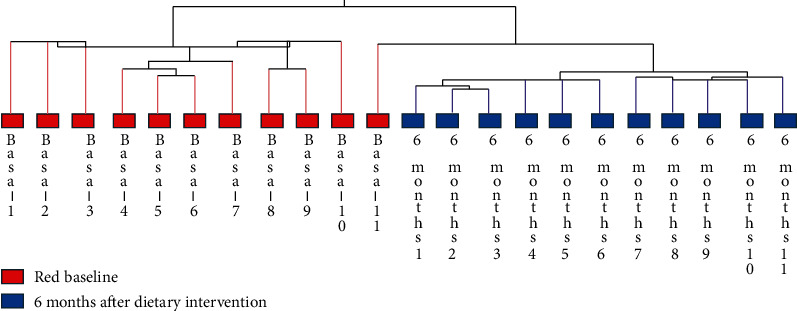
Supervised hierarchical clustering of obese patients, baseline vs. 6 months after dietary intervention.

Besides, when the total samples were evaluated, the hierarchical clustering evaluation shows that 10/11 baseline subjects cluster (Figure 3), only one subject was included in the second branch, and this subject had lower BMI and corporal weight (36.3 kg/m^2^ and 100.1 kg) than the remaining baseline subjects. The last group features all the 11 obese subjects before weight loss secondary to the hypocaloric Mediterranean diet.

Because hierarchical clustering demonstrated differences in gene profile levels of both branch and in order to avoid overlap amongst the metabolic pathways, we analyzed the associations of gene profile amongst and within the 2 situations. A gene group is defined as a number of genes with a similar purpose from the Gene Ontology project. All pathways have been analyzed with two algorithms (molecular and cell function) from GeneSpring, 13 categories have been reported (Table 2), and all the categories have been grouped in two the following: “endocrine system” and “nutritional disease.” Analysis of clusters reported 35 different genes in nutritional disease and 17 genes in endocrine system.

In the gene group of endocrine system disorder, the most important gene was thyroid peroxidase (TPO), this gene was overexpressed, and the next genes carbonic anhydrase VI (CA6), caveolin protein 1 (CAV1), and solute carrier family type 12 (SLLC12A3) were underexpressed. In the gene group of nutritional disease, the most relevant genes were carbonic anhydrase VI (CA6), caveolin protein 1 (CAV1), soluble carrier family type 12 (SLLC12A3), beta 3 receptor (ADRB3), and glutamate receptor ionotropic N methyl D aspartate 2 A (GRIN2A), and all of them were under expressed.

## 4. Discussion

Our analysis of PBMC gene expression by microarray in obese subjects after a hypocaloric Mediterranean diet showed a specific answer in the gene expression.

In the obesity treatment area, dietary changes such as different diets are the most important therapeutic tools to produce weight changes in obesity. Moreover, devices involved in DNA microarrays make easy to evaluate the role of a diet in the levels of expression of a lot of genes. Some studies with this idea report different gene expression following diets in human adipocytes [5, 26–28]. Moreover, a lack of studies in humans is detected, secondary to a problem to identify biomarkers that it is the accessibility to different tissues. Thus, finding a noninvasive source of RNA to evaluate the action of dietary interventions on gene profiler is an important area of investigation in nutrigenomic. Radich et al. [29] have shown the importance of the gene profile in PBMC to evaluate the differences in populations that may produce changes in treatment responses. For example, one study reported 35 genes downregulated in both adipocytes and PBMC from controls compared to obese subjects [30]. And our group showed that these blood cells in obese subjects presented 1436 differentially expressed genes compared with control subjects [31]. In other work [12], DNA microarrays showed that an 8-week hypocaloric diet without Mediterranean pattern produced to change weight is able to change an important diversity of biological pathways, such as metabolism, blood functions, immunological activity, and phosphorylation. In this previous design [12] and other similar ones [13, 14], the nutritional interventions have been caloric restriction without any determined diet.

One study [15] reported after a Mediterranean diet during 3 months supplemented with extravirgin olive oil and nuts a downregulation in neuroinflammation pathways in individuals with high cardiovascular risk. Castañer et al. [16] evaluated a subsample of PREDIMED study in high cardiovascular-risk patients, patients who received a diet with Mediterranean style supplemented with nuts for 3 months changed the expression of 241 genes, and those who received extravirgin olive changed the expression of 312 genes, and the most relevant pathways were associated to hypertension and atherosclerosis. Konstatntinidou et al. [17] demonstrated in a 3-month study in patients with high cardiovascular risk a modification of pathways related to oxidation and inflammation when using a diet with a Mediterranean pattern and olive oil. Our work was carried out in an obese population with low cardiovascular risk and with an intervention lasting 6 months, being able to explain the gene expression in different metabolic pathways that were observed. Undoubtedly, the type of patient, as well as the time of the dietary intervention and the type of diets, influences the results in the literature. For example, in the SYSDIET study with a Nordic isocaloric diet in patients with metabolic syndrome [32], modifications in gene expression were detected in inflammatory pathways. Some studies have demonstrated that Mediterranean-style diets include different dietary components [33, 34], which have been reported to exert beneficial biological effects, including antioxidant, anti-inflammatory, immunomodulatory, antitumoral, antilipemic, antidiabetic, and antiobesity activities. Mediterranean diet has shown positive effects on cardiovascular diseases, through the modulation of different gene expression, such as the following: the circadian locomotor output cycle protein kaput (CLOCK) gene [35] related with glucose metabolism and NF-*κ*B related with inflammation [36]. Interestingly, on the basis of different components of Mediterranean diet (omega 3 fatty acids, monounsaturated fatty acids, resveratrol, quercetin, and so on), it has been suggested that bioactive molecules in MedDiet may improve different cardiovascular risk factor through modulation of gene expression [37].

In our study with a low caloric diet with Mediterranean style in obese subjects without cardiovascular events or metabolic syndrome, results from clustering evaluation performed on the total of 634 genes were expressed in a different way with two principal pathways altered: cellular and metabolism functions. In these two main pathways, we reported a list of 262 upregulated expressed genes and 372 downregulated in obese patients after dietary intervention. Relevantly, we detected that highly upregulated genes were related in endocrine system, such as thyroid peroxidase (TPO), and the next genes carbonic anhydrase VI (CA6), caveolin protein 1 (CAV1), solute carrier family type 12 (SLLC12A3), beta 3 receptor (ADRB3), and glutamate receptor ionotropic N methyl D aspartate 2 A (GRIN2A) were under expressed. First, some lines of evidence had shown that adiposity increases the probability of having autoimmune thyroid diseases with an important role for a typical adipokine such as leptin as a peripheral determinant and thyroid peroxidase antibodies were more prevalent in the obese subjects [30]. Secondly, one finding in the literature was the high correlation between levels of CA6 and BMI in a group of females with obesity, which was not observed for a control group [38, 39]. Thirdly, caveolin 1 (CAV1) is a structural membrane protein that is composed of special cholesterol and sphingolipid-rich departments, above all in adipose tissue. For example, it has been shown that CAV1 is a relevant protein in sex hormone-dependent modulation of different pathways, for example, malignant tumors, obesity, and diabetes mellitus type 2. The CAV1 gene variant rs926198 is related with metabolic syndrome in different cohorts such as Caucasian and Hispanic [40], and other genetic variants have been associated with insulin resistance [41], too. Other findings suggested that CAV1 is related with the response of beta cell to different hypocaloric diets in animal models [42]. Fourthly, some studies showed that the gene encoding solute carrier family 12 member 3 (SLC12A3) is a typical candidate related with arterial hypertension in obese subjects, and its under expression could prevent this comorbidity in obese subjects [43]. Fifthly, in one study [44], the authors reported that ADRB3 DNA methylation in blood cells and adipocytes from visceral tissue was related with adiposity and its morbidities [44]. Lastly, the downregulation of GRIN2A in obese subjects could be associated with the tonic control of sympathetic pathway and arterial pressure [45], and the downregulation of it could be relevant in obesity to maintain blood pressure levels.

The actual pilot design has some strengths and limitations. The strength is that an intervention design has been realized to yield causality. Some weaknesses of our pilot design are associated to the sample size; only 11 obese subjects were recruited secondary to problems in detecting obese subjects without metabolic disturbances and drug treatments to comorbidities. Thus, other strategies evaluating microarray devices in metabolic diseases also have evaluated a similar number of individuals [5, 10–13]. Another limitation is the lack of a control group, without intervention or with a different diet. Finally, the intervention has the double aspect of a hypocaloric diet and the Mediterranean diet profile; therefore, both aspects may be influencing our results, and we cannot infer which is more important of the two in our findings.

Although the weaknesses expressed above, we reported that PBMCs from obese subjects without high cardiovascular risk after weight loss under a hypocaloric Mediterranean diet showed important modifications in gene profile, showing 634 genes with different expression. Besides, our findings reported a great number of genes related with some processes involved in metabolic pathways, with genes presenting up- and downregulation related with nutrition and endocrine system. Thus, our list of genes needs further investigation using these strategies which is aimed at developing more personalized hypocaloric diets with the knowledge of nutrigenomics in obese subjects without metabolic disturbances.

## Figures and Tables

**Table 1:** Changes in anthropometric and biochemical variables after a hypocaloric Mediterranean diet.

Parameters	Baseline	6 months	Baseline	6 months	Baseline	6 months
	Total group (*n* = 11)		Total group (*n* = 8) females		Total group (*n* = 3) males	
BMI (kg/m^2^)	38.6 ± 8.1	36.1 ± 5.1^∗^	38.2 ± 8.0	36.0 ± 4.1^∗^	38.7 ± 8.3	36.3 ± 4.2^∗^
Weight (kg)	106.1 ± 23.7	98.7 ± 18.3^∗^	105.1 ± 13.1	97.1 ± 15.3^∗^	107.9 ± 21.2	99.5 ± 13.3^∗^
Fat mass (kg)	45.9 ± 17.2	39.6 ± 14.1^∗^	45.0 ± 13.2	38.7 ± 12.1^∗^	46.4 ± 12.2	40.3 ± 19.0
WC (cm)	118.9 ± 16.4	113.1 ± 14.5^∗^	115.1 ± 11.4	110.0 ± 9.5^∗^	121.9 ± 19.1	114.1 ± 10.5^∗^
WHR	0.95 ± 0.08	0.93 ± 0.09^∗^	0.93 ± 0.06	0.91 ± 0.05^∗^	0.99 ± 0.08	0.95 ± 0.09^∗^
Glucose (mg/dl)	100.2 ± 9.5	98.2 ± 8.8	101.2 ± 9.2	99.3 ± 5.8	99.2 ± 9.1	98.0 ± 6.8
Total ch. (mg/dl)	194.5 ± 39.6	194.7 ± 38.3	193.5 ± 31.2	192.7 ± 21.3	194.9 ± 31.3	195.7 ± 28.3
LDL ch. (mg/dl)	110.5 ± 21.7	115.1 ± 24.8	109.5 ± 18.7	113.1 ± 13.8	111.2 ± 11.2	116.3 ± 21.8
HDL ch. (mg/dl)	54.7 ± 14.1	55.1 ± 9.8	55.7 ± 12.2	55.5 ± 9.1	54.3 ± 12.1	55.0 ± 7.8
TG (mg/dl)	128.5 ± 37.3	111.1 ± 27.4^∗^	126.1 ± 31.3	109.9 ± 21.2^∗^	131.9 ± 37.9	114.2 ± 28.8
CRP (mg/dl)	7.5 ± 5.1	5.4 ± 4.2^∗^	7.3 ± 5.0	5.1 ± 4.1^∗^	7.9 ± 5.4	5.9 ± 5.1
Insulin (mUI/L)	11.2 ± 4.2	9.1 ± 4.1^∗^	10.8 ± 4.1	8.7 ± 4.0^∗^	11.9 ± 4.9	9.7 ± 6.1
HOMA-IR (units)	2.4 ± 1.1	1.7 ± 0.6^∗^	2.2 ± 1.0	1.5 ± 0.3^∗^	2.7 ± 1.3	2.0 ± 0.9

BMI: body mass index; ch: cholesterol; TG: triglycerides; CRP: C-reactive protein; HOMA-IR: homeostasis model assessment. ^∗^*p* < 0.05 means statistical differences after dietary intervention. Paired Student's *t*-tests for parametric parameters and nonparametric variables were analyzed with the Mann-Whitney *U* test.

**Table 2:** Nutritional disease and endocrine system.

Endocrine system	*p* value
Hypercalciuria	3.08*E*-03
Hypocalciuria	5.66*E*-03
Thyroid dyshormonogenesis	7.46*E*-03
Follicular thyroid cancer	1.15*E*-02
Bartter syndrome type 3 with hypocalciuria	1.69*E*-02
Perlman syndrome	1.69*E*-02
Cytochrome P450 oxidoreductase deficiency	1.69*E*-02
Deficiency of iodide peroxidase	1.69*E*-02
Nutritional disease	
Hyperphagia	1.38*E*-04
Obesity	1.88*E*-03
Body mass index	1.25*E*-02
Hyponatremia of malignancy	1.69*E*-02
Hyperphagia	1.38*E*-04

*p* value: measure of the likelihood that the association between a set of genes in your dataset and a related function is due to random association. The smaller the *p* value, the less likely that the association is random and the more significant the association. Fisher test.

## Data Availability

Data available on request through the corresponding author Prof. Dr. Daniel de Luis (dadluis@yahoo.es).

## Abbreviations

TPO: Thyroid peroxidase
CA6: Carbonic anhydrase VI
CAV1: Caveolin protein 1
SLLC12A3: Solute carrier family type 12
ADRB3: Beta 3 receptor
GRIN2A: Glutamate receptor ionotropic N methyl D aspartate 2 A
PBMCs: Peripheral blood mononuclear cells.

## References

[1] Guh D. P., Zhang W., Bansback N., Amarsi Z., Birmingham C. L., Anis A. H. (2009). The incidence of co-morbidities related to obesity and overweight: a systematic review and meta-analysis. *BMC Public Health*.

[2] Loos R. J., Bouchard C. (2003). Obesity–is it a genetic disorder?. *Journal of Internal Medicine*.

[3] Heidecker B., Hare J. M. (2007). The use of transcriptomic biomarkers for personalized medicine. *Heart Failure Reviews*.

[4] Hernandez-Morante J. J., Milagro F. I., Lujan J. A., Martínez J. A., Zamora S., Garaulet M. (2008). Insulin effect on adipose tissue (AT) adiponectin expression is regulated by the insulin resistance status of the patients. *Clinical Endocrinology*.

[5] Baranova A., Collantes R., Gowder S. J. (2005). Obesity-related differential gene expression in the visceral adipose tissue. *Obesity Surgery*.

[6] Kussmann M., Raymond F., Affolter M. (2006). OMICS-driven biomarker discovery in nutrition and health. *Journal of Biotechnology*.

[7] DePrimo S. E., Wong L. M., Khatry D. B. (2003). Expression profiling of blood samples from an SU5416 phase III metastatic colorectal cancer clinical trial: a novel strategy for biomarker identification. *BMC Cancer*.

[8] Horwitz P. A., Tsai E. J., Putt M. E. (2004). Detection of cardiac allograft rejection and response to immunosuppressive therapy with peripheral blood gene expression. *Circulation*.

[9] Liew C. C., Ma J., Tang H. C., Zheng R., Dempsey A. A. (2006). The peripheral blood transcriptome dynamically reflects system wide biology: a potential diagnostic tool. *The Journal of Laboratory and Clinical Medicine*.

[10] Bouwens M., Grootte B. M., Jansen J., Muller M., Afman L. A. (2010). Postprandial dietary lipid-specific effects on human peripheral blood mononuclear cell gene expression profiles. *The American Journal of Clinical Nutrition*.

[11] Bouwens M., van de Rest O., Dellschaft N. (2009). Fish-oil supplementation induces antiinflammatory gene expression profiles in human blood mononuclear cells. *The American Journal of Clinical Nutrition*.

[12] Crujeiras A. B., Parra D., Milagro F. I. (2008). Differential expression of oxidative stress and inflammation related genes in peripheral blood mononuclear cells in response to a low-calorie diet: a nutrigenomics study. *OMICS*.

[13] Mangravite L. M., Dawson K., Davis R. R., Gregg J. P., Krauss R. M. (2007). Fatty acid desaturase regulation in adipose tissue by dietary composition is independent of weight loss and is correlated with the plasma triacylglycerol response. *The American Journal of Clinical Nutrition*.

[14] Brattbakk H. R., Arbo I., Aagaard S. (2013). Balanced caloric macronutrient composition downregulates immunological gene expression in human blood cells adipose tissue diverges. *OMICS*.

[15] Almanza-Aguilera E., Hernáez A., Corella D. (2020). Transcriptional response to a Mediterranean diet intervention exerts a modulatory effect on neuroinflammation signaling pathway. *Nutritional Neuroscience*.

[16] Castañer O., Corella D., Covas M. I. (2013). In vivo transcriptomic profile after a Mediterranean diet in high-cardiovascular risk patients: a randomized controlled trial. *The American Journal of Clinical Nutrition*.

[17] Konstantinidou V., Covas M. I., Muñoz-Aguayo D. (2010). In vivo nutrigenomic effects of virgin olive oil polyphenols within the frame of the Mediterranean diet: a randomized controlled trial. *The FASEB Journal*.

[18] Mancini J. G., Filion K. B., Atallah R. (2016). Systematic review of the Mediterranean diet for long-term weight loss. *The American Journal of Medicine*.

[19] Siitonen N., Pulkkinen L., Lindström J. (2011). Association of ADIPOQ gene variants with body weight, type 2 diabetes and serum adiponectin concentrations: the Finnish Diabetes Prevention Study. *BMC Medical Genetics*.

[20] Widmer R. J., Flammer A. J., Lerman L. O., Lerman A. (2015). The Mediterranean diet, its components, and cardiovascular disease. *The American Journal of Medicine*.

[21] Expert Panel on Detection, Evaluation, and Treatment of High Blood Cholesterol in Adults (2001). Expert panel on detection, evaluation, and treatment of high blood cholesterol in adults: executive summary of the third report of the National Cholesterol Education Program (NCEP) expert panel on detection, evaluation, and treatment of high blood cholesterol in adults: (adult treatment panel III). *Journal of the American Medical Association*.

[22] Mataix J., Mañas M. (2003). *Tablas de Composición de Alimentos Españoles*.

[23] Friedewald W. T., Levy R. J., Fredrickson D. S. (1972). Estimation of the concentration of low-density lipoprotein cholesterol in plasma without use of the preparative ultracentrifuge. *Clinical Chemistry*.

[24] Mathews D. R., Hosker J. P., Rudenski A. S., Naylor B. A., Df T. (1985). Homeostasis model assessment: insulin resistance and ?-cell function from fasting plasma glucose and insulin concentrations in man. *Diabetologia*.

[25] Lukaski H., Johson P. E. (1985). Assessment of fat-free mass using bioelectrical impedance measurements of the human body. *The American Journal of Clinical Nutrition*.

[26] Ashburner M., Ball C. A., Blake J. A. (2000). Gene Ontology: tool for the unification of biology. *Nature Genetics*.

[27] ClÉment K., Viguerie N., Poitou C. (2004). Weight loss regulates inflammation-related genes in white adipose tissue of obese subjects. *FASEB*.

[28] Viguerie N., Poitou C., Cancello R., Stich V., Clement K., Langin D. (2005). Transcriptomics applied to obesity and caloric restriction. *Biochimie*.

[29] Radich J. P., Mao M., Stepaniants S. (2004). Individual-specific variation of gene expression in peripheral blood leukocytes. *Genomics*.

[30] García-Amigot F. L. O., Marti A., Moreno-Aliaga M. J., Bandrés E., García-Foncillas J., Martínez J. A., Ling P. R. (2005). Gene Expression Pattern of Obese and Control Individuals in Adipose Tissue and Peripheral Blood Mononuclear Cells. *Trends in Obesity Research*.

[31] de Luis D. A., Almansa R., Aller R., Izaola O., Romero E. (2018). Gene expression analysis identify a metabolic and cell function alterations as a hallmark of obesity without metabolic syndrome in peripheral blood, a pilot study. *Clinical Nutrition*.

[32] Ulven S. M., Holven K. B., Rundblad A. (2019). An isocaloric nordic diet modulates RELA and TNFRSF1A gene expression in peripheral blood mononuclear cells in individuals with metabolic syndrome-a SYSDIET sub-study. *Nutrients*.

[33] Peyrol J., Riva C., Amiot M. (2017). Hydroxytyrosol in the prevention of the metabolic syndrome and related disorders. *Nutrients*.

[34] Vinson J. A., Cai Y. (2012). Nuts, especially walnuts, have both antioxidant quantity and efficacy and exhibit significant potential health benefits. *Food & Function*.

[35] Corella D., Asensio E. M., Coltell O. (2016). CLOCK gene variation is associated with incidence of type-2 diabetes and cardiovascular diseases in type-2 diabetic subjects: dietary modulation in the PREDIMED randomized trial. *Cardiovascular Diabetology*.

[36] Ghanim H., Aljada A., Hofmeyer D., Syed T., Mohanty P., Dandona P. (2004). Circulating mononuclear cells in the obese are in a proinflammatory state. *Circulation*.

[37] Toledo E., Wang D. D., Ruiz-Canela M. (2017). Plasma lipidomic profiles and cardiovascular events in a randomized intervention trial with the Mediterranean diet. *The American Journal of Clinical Nutrition*.

[38] Marzullo P., Minocci A., Tagliaferri M. A. (2010). Investigations of Thyroid Hormones and Antibodies in Obesity: Leptin Levels Are Associated with Thyroid Autoimmunity Independent of Bioanthropometric, Hormonal, and Weight-Related Determinants. *The Journal of Clinical Endocrinology and Metabolism*.

[39] Lamy E., Simões C., Rodrigues L. (2015). Changes in the salivary protein profile of morbidly obese women either previously subjected to bariatric surgery or not. *Journal Of Physiology And Biochemistry*.

[40] Baudrand R., Goodarzi M. O., Vaidya A. (2015). A prevalent caveolin-1 gene variant is associated with the metabolic syndrome in Caucasians and Hispanics. *Metabolism*.

[41] Abaj F., Saeedy S. A. G., Mirzaei K. (2021). Are caveolin-1 minor alleles more likely to be risk alleles in insulin resistance mechanisms in metabolic diseases?. *BMC Research Notes*.

[42] Lillo Urzúa P., Núñez Murillo O., Castro-Sepúlveda M. (2020). Loss of caveolin-1 is associated with a decrease in beta cell death in mice on a high fat diet. *International Journal of Molecular Sciences*.

[43] Wang Y. L., Qi Y., Bai J. N. (2014). Tag polymorphisms of solute carrier family 12 member 3 gene modify the risk of hypertension in northeastern Han Chinese. *Journal of Human Hypertension*.

[44] Guay S. P., Brisson D., Lamarche B. (2014). ADRB3 gene promoter DNA methylation in blood and visceral adipose tissue is associated with metabolic disturbances in men. *Epigenomics*.

[45] Cui B. P., Li P., Sun H. J. (2013). Ionotropic glutamate receptors in paraventricular nucleus mediate adipose afferent reflex and regulate sympathetic outflow in rats. *Acta Physiologica*.

